# Selective Affimers Recognise the BCL‐2 Family Proteins BCL‐x_L_ and MCL‐1 through Noncanonical Structural Motifs**

**DOI:** 10.1002/cbic.202000585

**Published:** 2020-11-02

**Authors:** Jennifer A. Miles, Fruzsina Hobor, Chi H. Trinh, James Taylor, Christian Tiede, Philip R. Rowell, Brian R. Jackson, Fatima A. Nadat, Pallavi Ramsahye, Hannah F. Kyle, Basile I. M. Wicky, Jane Clarke, Darren C. Tomlinson, Andrew J. Wilson, Thomas A. Edwards

**Affiliations:** ^1^ School of Molecular and Cellular Biology University of Leeds Woodhouse Lane Leeds LS2 9JT UK; ^2^ Astbury Centre For Structural Molecular Biology University of Leeds Woodhouse Lane Leeds LS2 9JT UK; ^3^ School of Chemistry University of Leeds Woodhouse Lane Leeds LS2 9JT UK; ^4^ Protein Production Facility University of Leeds Woodhouse Lane Leeds LS2 9JT UK; ^5^ Department of Chemistry University of Cambridge Lensfield Road Cambridge CB2 1EW UK

**Keywords:** Affimers, BCL-2 family, chemical biology, non-antibody-binding proteins, protein-protein interactions

## Abstract

**Abstract**: The BCL‐2 family is a challenging group of proteins to target selectively due to sequence and structural homologies across the family. Selective ligands for the BCL‐2 family regulators of apoptosis are useful as probes to understand cell biology and apoptotic signalling pathways, and as starting points for inhibitor design. We have used phage display to isolate Affimer reagents (non‐antibody‐binding proteins based on a conserved scaffold) to identify ligands for MCL‐1, BCL‐x_L_, BCL‐2, BAK and BAX, then used multiple biophysical characterisation methods to probe the interactions. We established that purified Affimers elicit selective recognition of their target BCL‐2 protein. For anti‐apoptotic targets BCL‐x_L_ and MCL‐1, competitive inhibition of their canonical protein‐protein interactions is demonstrated. Co‐crystal structures reveal an unprecedented mode of molecular recognition; where a BH3 helix is normally bound, flexible loops from the Affimer dock into the BH3 binding cleft. Moreover, the Affimers induce a change in the target proteins towards a desirable drug‐bound‐like conformation. These proof‐of‐concept studies indicate that Affimers could be used as alternative templates to inspire the design of selective BCL‐2 family modulators and more generally other protein‐protein interaction inhibitors.

## Graphical abstract

**Affimer reagents** (non‐antibody binding proteins) are identified that bind selectively to the anti‐apoptotic proteins BCL‐x_L_ and MCL‐1. Crystallographic studies reveal an unprecedented mode of molecular recognition in which flexible loops from the Affimer dock into the BH3 binding cleft as opposed to a canonical α‐helix. Furthermore the Affimers induce changes in the target proteins towards desirable drug‐bound‐like conformations.

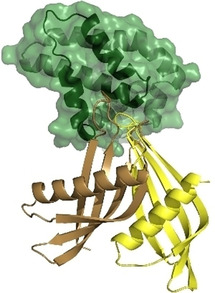

## Introduction

**Figure 1:**
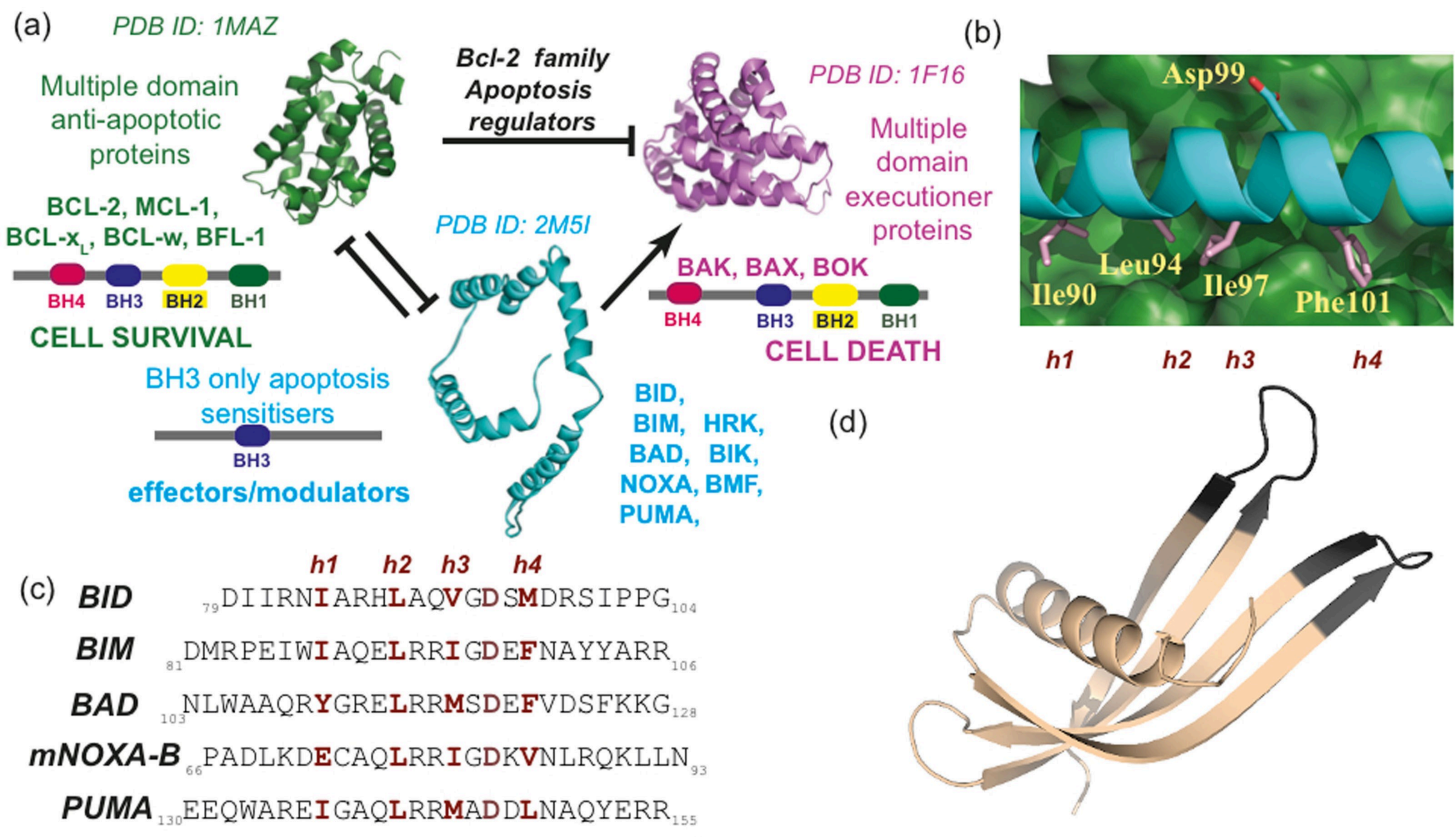
BCL‐2 family structure and function. a) Schematic annotating the BCL‐2 family member role in apoptosis. Activation of the executioner proteins (purple) triggers apoptosis; this depends on the balance of anti‐apoptotic proteins (green) vs. effectors (blue).[^47^, ^48^, ^49^] b) BCL‐x_L_(green)/BIM(cyan) co‐crystal structure (PDB ID: 1PQ1) highlighting key residues for binding (pink for hydrophobic h1–h4 positions and key aspartic acid labelled above and below). c) Sequences of BH3 modulators (key residues required for BH3 cleft affinity in dark red). d) Crystal structure of an Affimer highlighting variable regions (dark grey) where amino acid variation is possible (PDB ID: 5A0O).^[38]^

A central challenge in life sciences research is to identify modulators of protein‐protein interactions (PPIs).[^1^, ^2^] Such modulators represent probes with which to uncover new understanding of structural and cellular biology, as well as starting points for drug discovery. The BCL‐2 family of PPIs are an important class of α‐helix‐mediated interaction that control the intrinsic apoptosis pathway.^[3]^ Their critical role in apoptosis has prompted efforts to identify modulators so as to facilitate greater understanding of both BCL‐2 family signalling and drug discovery.[^4^, ^5^, ^6^, ^7^, ^8^] Moreover differing selectivities and specificities amongst BCL‐2 family member interactions[^9^, ^10^] render the family an outstanding model system to elaborate novel generic chemical and biological approaches for protein‐protein interaction modulation.[^11^, ^12^] BCL‐2 family proteins can be identified through their BCL‐homology (BH) domains and may be categorised within three specific sub‐groups (Figure 1a). Pro‐apoptotic (or executioner) proteins such as BAK and BAX activate apoptosis through pore formation in the mitochondrial membrane; anti‐apoptotic proteins including BCL‐2, MCL‐1 and BCL‐x_L_, sequester pro‐apoptotic members to prevent cell death; and a group of regulatory proteins which bind to other BCL‐2 members (including BIM, BID, BAD, NOXA and PUMA), mediate initiation of apoptosis. In all cases binding between BCL‐2 family members occurs through the BH3 homology domain of one protein, which forms an α‐helix upon binding and docks into a complementary cleft on its partner (Figure 1b). The BH3 ligand exploits conserved hydrophobic residues in positions *i*, *i*+4, *i*+7 and *i*+11 together with a conserved aspartic acid (at *i*+9) to achieve high affinity interaction with the BH3 cleft (Figure 1c). *In silico* and experimental approaches have been used to identify selective sequences for individual BCL‐2 family members.[^13^, ^14^, ^15^, ^16^] Multiple studies have endeavoured to identify chemotypes which mimic the BH3 domains so as to orthosterically inhibit BCL‐2/BH3 PPIs including: constrained peptides,[^17^, ^18^, ^19^, ^20^, ^21^, ^22^, ^23^] peptidomimetics,[^24^, ^25^, ^26^, ^27^] small molecules[^28^, ^29^, ^30^, ^31^] and miniature proteins (identified with assistance from biological selection).[^32^, ^33^] In this work we have used a previously described *Affimer* library[^34^, ^35^, ^36^, ^37^, ^38^, ^39^] to identify ligands for MCL‐1, BCL‐x_L_, BCL‐2, BAK and BAX and selective inhibitors of MCL‐1 and BCL‐x_L_ interactions with cognate BH3 partners. Our aim was not only to identify high affinity binders, but also to then screen for subsets that would inhibit PPIs, provide multiple sequences for motif identification, and to use those amenable to structural studies to understand the mode of binding. Affimer reagents belong to an emerging class of non‐antibody‐based protein scaffolds which include, Monobodies, Darpins, Affibodies and others,[^40^, ^41^, ^42^, ^43^, ^44^] which might offer advantages in therapeutic and diagnostic settings associated with improved solubility, purification, expression and stability. Affimer reagents are based on either a human scaffold,^[45]^ or a phytocystatin scaffold (Figure 1d) which has been optimised by homology.^[39]^ Both show high thermal stability and achieve molecular recognition through one or two variable regions (VRs) of between six and twelve amino acid residues. Multiple large libraries of Affimers have been established permitting biological selection of optimised binding reagents through randomisation within each of the VRs.[^39^, ^46^] These reagents provide access to distinct compositional and conformational peptide diversity compared to natural biological peptides, and can be identified *via* the power of genetics rather than synthetic chemistry.

## Results

### Isolating Affimers

Following expression using established methods (see the Supporting Information), MCL‐1, BCL‐x_L_, BCL‐2, BAK and BAX were biotinylated and immobilised on plates, over which the library of Affimers was panned in order to isolate high‐affinity binders (see the Supporting Information). Phage ELISA was then used to identify clones that bind selectively for further analysis. Following this screening, candidate Affimer reagents were sequenced resulting in twelve unique sequences with affinity for MCL‐1 (from 24 clones), eleven for BCL‐x_L_ (21 clones), four for BCL‐2 (20 clones), five for BAX (31 clones) and four for BAK (24 clones). Tables S1 and S2 in the Supporting Information indicate the identified sequences and frequency.

### Binding analysis of Affimers

**Figure 2:**
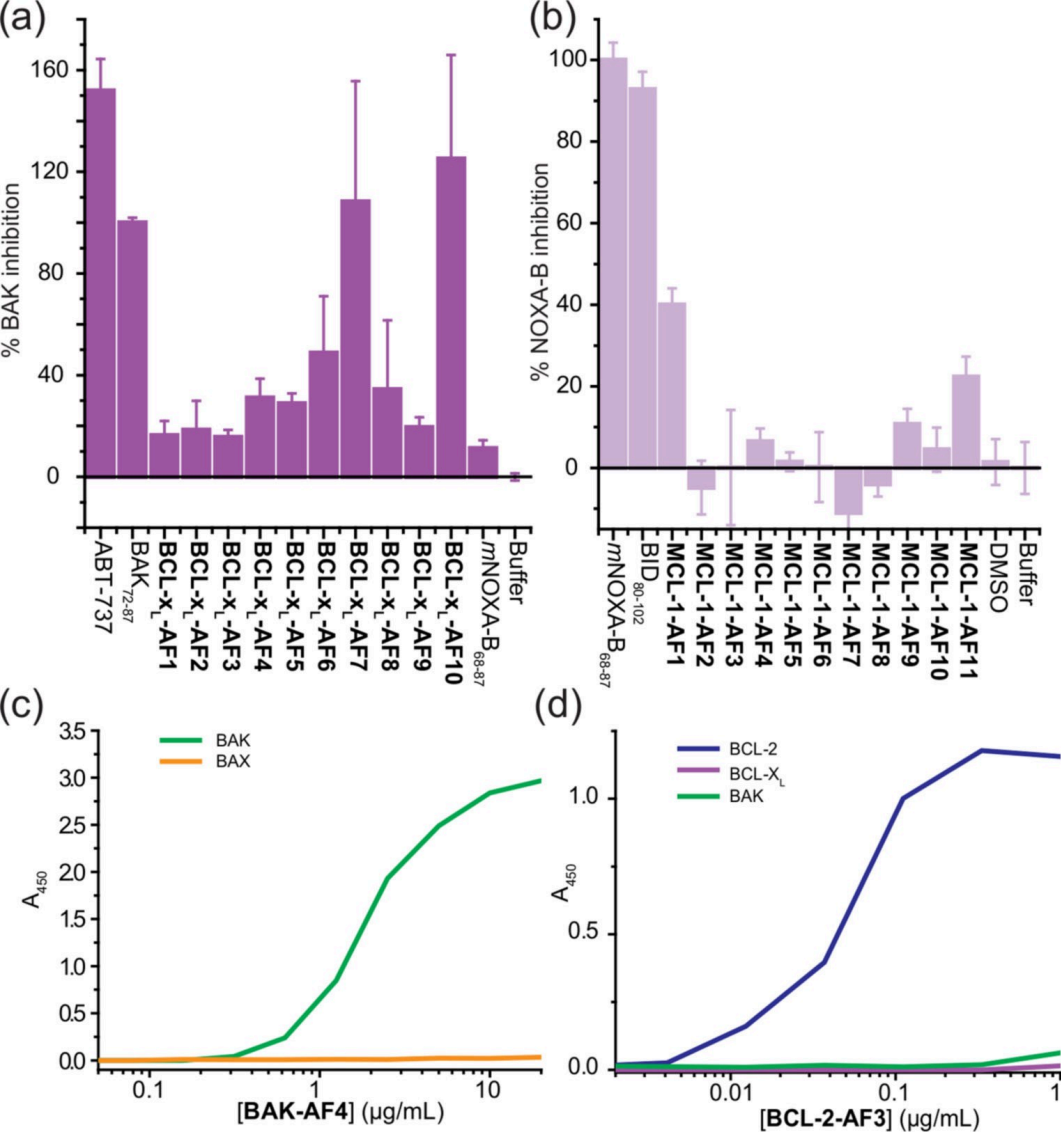
Binding analysis and selectivity of BCL‐2 family binding Affimers. Single‐point screening of a) BCL‐x_L_ binding Affimers (1 μM, *n*=3) for competitive inhibition of BH3 binding and b) MCL‐1 binding Affimers (1 μM, *n*=3. Binding ELISA for c) BAK‐AF4 and d) BCL‐2‐AF3. Error bars represent SD.

Small‐scale expression of the Affimers allowed preliminary biophysical/biochemical analyses. For MCL‐1 and BCL‐x_L_, single concentration fluorescence anisotropy (FA) competition assays (Figure 2a, b) against BCL‐x_L_/BODIPY‐Ahx‐BAK_72–87_ or MCL‐1/FITC‐Ahx‐*m*NOXA‐B_68–87_ (using competitor Affimer at 1 μM) were used to identify Affimers that inhibit cognate BH3 binding. Inhibition was compared to positive controls BAK_72–87_ and ABT‐737 for BCL‐x_L_ and *m*NOXA‐B_68–87_ for MCL‐1 with the peptide activity defined as 100 % inhibition (ABT‐737 is more active than BAK therefore achieves 150 % inhibition). From these assays, three BCL‐x_L_ Affimers were identified with significant inhibitory potency, (BCL‐x_L_‐AF6, BCL‐x_L_‐AF7 and BCL‐x_L_‐AF10) and two MCL‐1 Affimers (MCL‐1‐AF1 and MCL‐1‐AF11). It should be noted that the screening process (for all of the BCL‐2 family targets), may have selected for Affimers that do not recognise the BH3 cleft, so a degree of attrition is to be expected. We did not have an established competition assay for BCL‐2, BAK and BAX. BAK, BAX and BCL‐2 Affimers were therefore purified by size‐exclusion chromatography and confirmation of correct Affimer folding was obtained through circular dichroism (CD, see the Supporting Information, Figure S1). Binding ELISA using a primary antibody for the His tag on the Affimer and secondary HRP antibody established selective interaction between the Affimer and BCL‐2 targets (Figures 2 c, d and S2): BCL‐2‐AF1 to BCL‐2‐AF3 and BAK‐AF1 to BAK‐AF4 were confirmed as genuine binders, selective for their targets, but no BAX Affimers were successfully confirmed from the ELISA analyses. This may arise due to nonspecific and/or weak binding of Affimers towards BAX. Ultimately, the competitive inhibitors of BCL‐x_L_ and MCL‐1 identified from these preliminary screens were considered sufficient for more detailed studies exploring the structural and thermodynamic bases of molecular recognition.

### Biophysical analysis of Affimers

**Figure 3:**
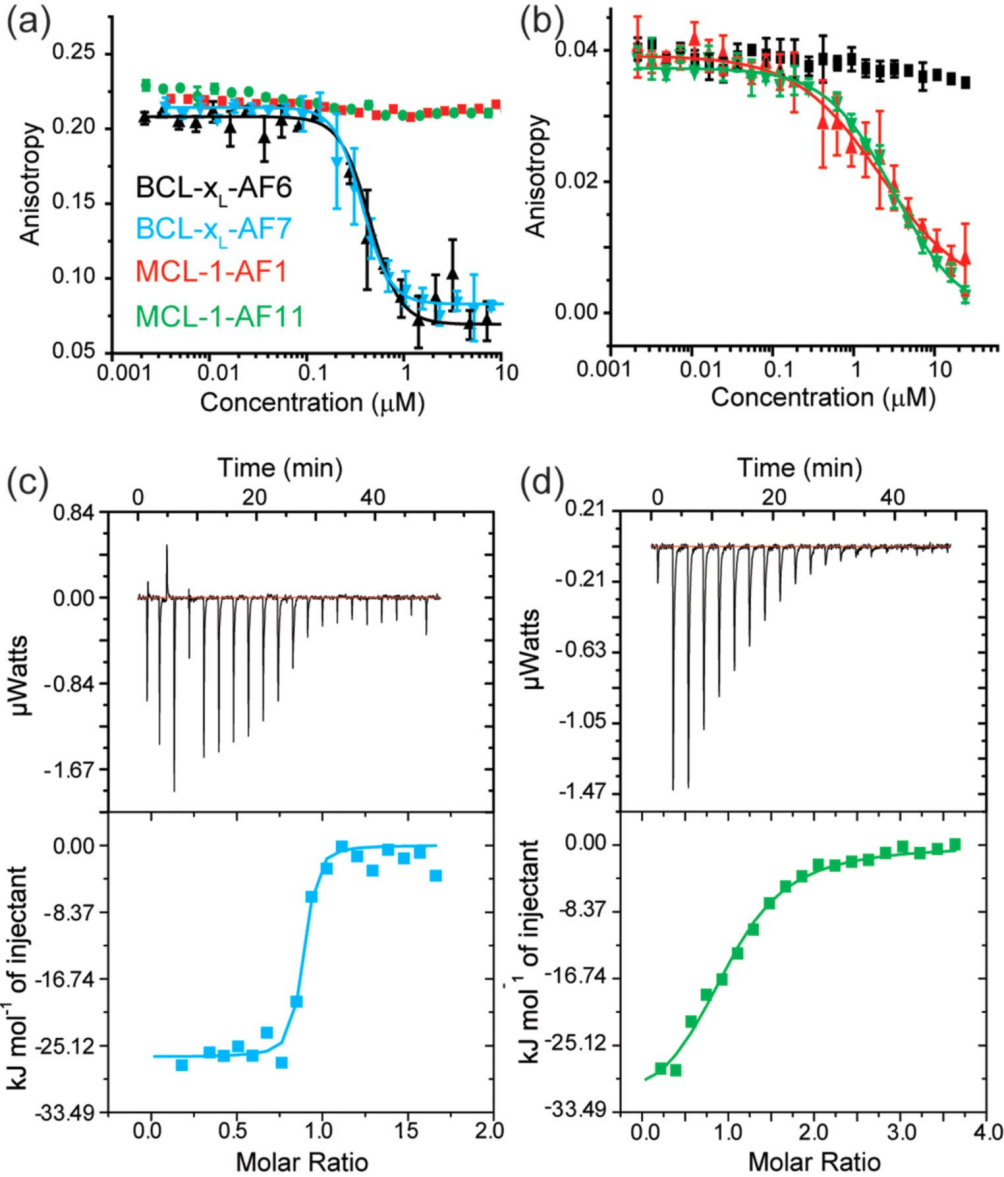
Biophysical analyses of BCL‐x_L_ and MCL‐1 binding Affimers. Fluorescence anisotropy competition assays (*n*=3) for a) BCL‐x_L_/BODIPY‐Ahx‐BAK_72–87_ and b) MCL‐1/FITC‐Ahx‐*m*NOXA‐B_68–87_. ITC data for binding of Affimers to BCL‐x_L_ and MCL‐1, thermograms and curve fitting for ITC analyses on the c) BCL‐x_L_‐AF7/BCL‐x_L_ and d) MCL‐1‐AF11/MCL‐1 interactions; colour coding as for (a).

Larger‐scale expression in *E. coli* of the five Affimers identified from single‐point FA competition allowed the purified proteins to be tested in full dose response fluorescence anisotropy competition assays against their target (Figure 3a, b). BCL‐x_L_‐AF10 showed problems during purification so was not further characterised. Both BCL‐x_L_‐AF6 (IC_50_=448±53 nM) and BCL‐x_L_‐AF7 (IC_50_=393 ±54 nM) were shown to act as sub μM inhibitors of the BCL‐x_L_/BAK interaction (Figure 3a) but were ineffective in inhibiting the MCL‐1/NOXA−B interaction. Similarly, the Affimers selected for MCL‐1 binding were shown to act as low μM inhibitors of their target interaction (MCL‐1‐AF1 IC_50_=2.1±0.2 μM; MCL‐1‐AF11 IC_50_=3.2±0.4 μM) but did not inhibit BCL‐x_L_ /BAK, (Figure 3b), demonstrating selectivity.

Isothermal titration calorimetry (ITC; Figures 3c, d and S3, Table 1) confirmed the conclusions garnered from competition FA and gave *K*_d_ values consistent with the determined IC_50_ values. Both BCL‐x_L_‐AF6 (BCL‐x_L_ selective) and MCL‐1‐AF11 (MCL‐1 selective) Affimers exhibited favourable enthalpic but unfavourable entropic contributions to binding (it was not possible to obtain data for MCL‐AF1). In the case of BCL‐x_L_‐AF6 a particularly strong enthalpic contribution was observed. On the other hand BCL‐x_L_‐AF7 was found to be favourable in both the enthalpic and entropic terms. Given the hydrophobic nature of the BH3 binding cleft and high conservation of aliphatic side chains at key positions in both BH3 sequences and the Affimers (see discussion of co‐crystal structure below), the observation that different thermodynamic signatures can be employed to achieve recognition could be a useful consideration in informing inhibitor design. Whilst thermodynamic signatures are notoriously difficult to interpret, and enthalpically driven hydrophobic molecular recognition has been documented, the “classical” view of hydrophobic driven binding is one of entropic desolvation.[^50^, ^51^, ^52^, ^53^] Moreover, our own prior studies characterised BH3/BCL‐2 family interactions as entropically driven.^[20]^

**Table 1:** Thermodynamic parameters of Affimer/BCL‐x_L_ and Affimer/MCL‐1 binding determined by ITC.

Protein	Ligand	*K*_d_ [nM]	Δ*H* [kJ/mol]	*T*Δ*S* [J/mol]
BCL‐x_L_	BCL‐x_L_‐AF6	90.9±3.0	−45.3±0.93	−17.1
BCL‐x_L_	BCL‐x_L_‐AF7	38.6±1.0	−26.5±0.75	53.0
MCL‐1	MCL‐1‐AF11	3400±400	−34.4±1.76	−11.34

Whilst the Affimer technology regularly produces binders with *K*_d_ in the nanomolar range,^[36]^ here we added multiple layers of screening (inhibition of BH3 binding; compatible with anisotropy and ITC experiments) in addition to panning for high affinity binders. This will naturally lead to an attrition rate where clones that do not meet the criteria are lost, which may explain the slightly lower affinities we observe (Table 1). In this study, we chose this approach as the selectivity achieved here is of significantly greater value than affinity alone in experiments where inhibition of a single member of a highly homologous family is desired. With additional and more stringent panning and selection (potentially quicker), or a larger library or use of affinity maturation techniques (slower), better affinities may be achievable where all criteria are met.

### Crystal structures and conformational selection

Having established that Affimers act as selective inhibitors of BCL‐2 family PPIs, we attempted to obtain co‐crystals to allow high‐resolution structural interpretation of the interactions. BCL‐x_L_‐AF6/BCL‐x_L,_ BCL‐x_L_‐AF7/BCL‐x_L_ and MCL‐1‐AF11/MCL‐1 co‐crystals were obtained (see the Supporting Information, and Table S3); and the structures solved by molecular replacement using Phaser.^[54]^

**Figure 4:**
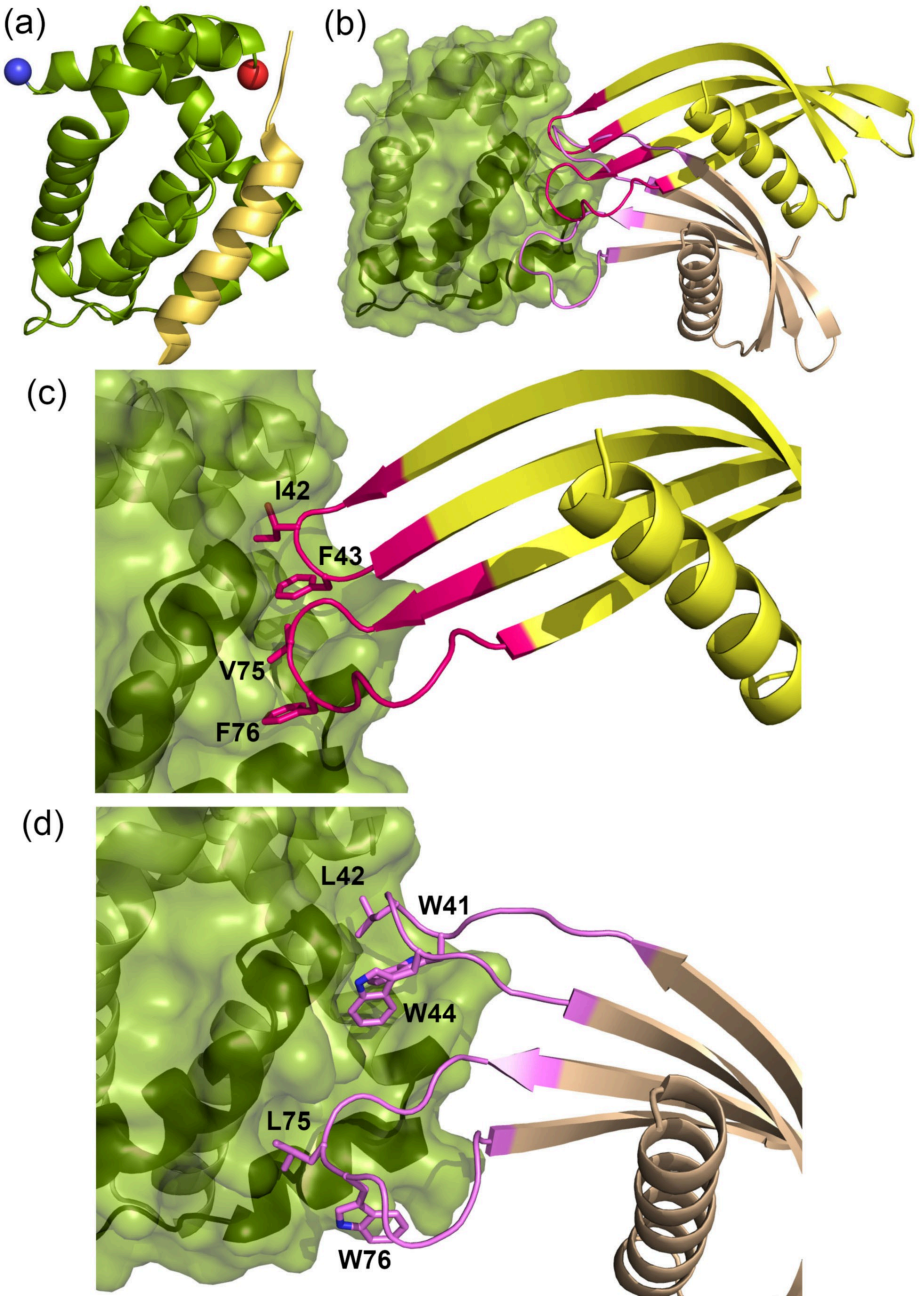
Affimer: BCL‐x_L_ co‐crystal structures. a) Cartoon representation of Bcl‐x_L_ bound to BAK peptide (PDB ID: 5FMK), in the same orientation in all panels (the blue sphere is at the N terminus, red sphere C terminus). b) Overlay of Affimers BCL‐x_L_‐AF6 (yellow) and BCL‐x_L_‐AF7 (brown) bound to BCL‐x_L_ (green) illustrating the projection of Affimer loops into the BH3 binding cleft. c) Enlargement of hydrophobic clusters from VRs 1 and 2 of BCL‐x_L_‐AF6 (magenta side chains, labelled) projecting into the BH3 cleft. d) Hydrophobic cluster from VRs 1 and 2 of BCL‐x_L_‐AF7 (purple side chains, labelled). Both Affimers bind in the cleft, but at slightly different sites and using different side chains.

For BCL‐x_L_‐AF6/BCL‐x_L,_ the crystals diffracted to 1.91 Å, and the asymmetric unit contains one domain swapped dimer of BCL‐x_L_, with one BCL‐x_L_‐AF6 bound to the cleft of each monomer (Figure 4b, c). For BCL‐x_L_‐AF7/BCL‐x_L,_ the crystals diffract to 2.24 Å, and the asymmetric unit contains two domain swapped dimers of BCL‐x_L_, with one BCL‐x_L_‐AF7 bound to the cleft of each monomer (Figure 4b, d). The residues within the VRs of both Affimers interact with residues lining the BH3‐binding cleft on the surface of BCL‐x_L_. Representative electron density is presented in Figure S4. As expected, given that we selected for competitive Affimers, the Affimers bind at the BH3 binding groove. Indeed, the Affimers use some of the available binding pockets in the groove. In BCL‐x_L_‐AF6, F43 binds to the pocket as does F101 on BIM (in PDB 5C3G), and F76 binds the same pocket as I97 on BIM. For BCL‐x_L_‐AF7, W41 binds the same pocket as F101 on BIM. However, the universally conserved BH3 Asp which hydrogen‐bonds to Arg on BCL2‐ family members is not replicated in any way by the Affimer.

**Figure 5:**
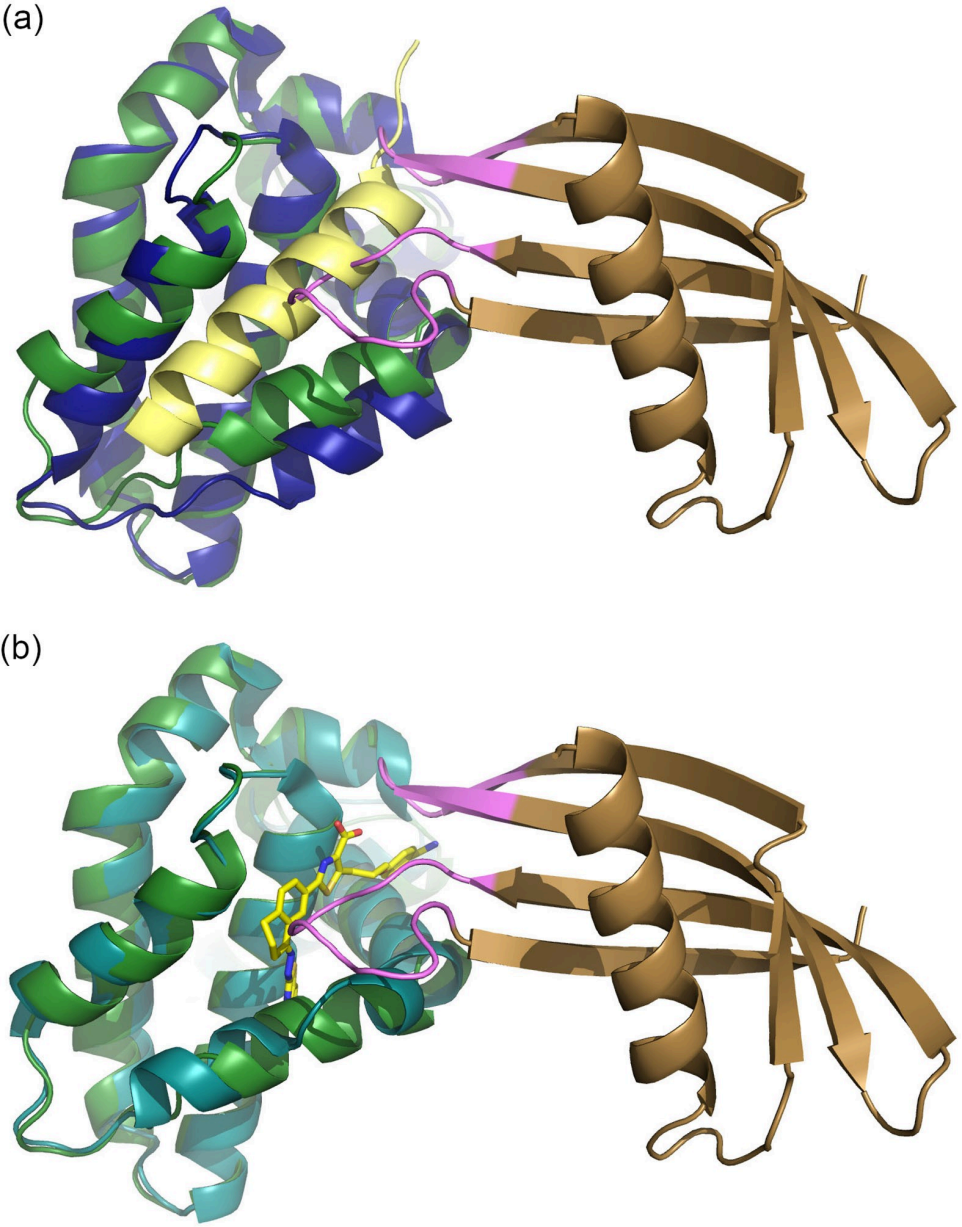
Conformational selection of BCL‐x_L_ by the Affimers. When bound to Affimer (BCL‐x_L_‐AF6 or BCL‐x_L_‐AF7), BCL‐x_L_ is in the small‐molecule‐bound conformation, not the peptide‐bound conformation. a) Superposition of BCL‐x_L_ when bound to BCL‐x_L_‐AF7 (BCL‐x_L_ dark green, BCL‐x_L_‐AF7 brown) vs. BAK (PDB ID: 5FMK; BCL‐x_L_ blue, BAK peptide yellow) reveals a change in conformation at the peptide binding groove (note the different position of the helix towards the bottom of the panel). b) Superposition of BCL‐x_L_ when bound to BCL‐x_L_‐AF7 (dark green) vs. WEHI‐539 (PDB ID: 3ZLR; BCL‐x_L_ cyan, compound in yellow) where the groove is in the same conformation (note the overlay of the helices). The helix binding groove is 5 Å wider at the last turn when bound to peptide than to WEHI‐539 or BCL‐x_L_‐AF7 (14.8 vs. 10–11 Å). This might be a favourable conformation to select for protein‐protein interaction inhibition.

On inspection it is apparent that the Affimers select a distinct conformation of the BCL‐x_L_ domain. The binding groove on BCL‐x_L_ is formed by helices 3 and 4 (Figure 5) and helix 3 is mobile such that the width of the groove can vary. When BH3 peptide is bound, helix 3 moves to accommodate the peptide in a relatively wide groove (groove open conformation). By comparison, when BCL‐x_L_ is bound to the small molecule ABT‐737, and other small molecules, such as WEHI‐539 (Table S4),^[55]^ or to BCL‐x_L_‐AF6 and BCL‐x_L_‐AF7, the groove is narrower (Figure 5). When bound to BAK peptide the groove is 14.8 Å wide at the widest point (Figure 5a), whereas in the small molecule and Affimer bound conformation it is ∼11.0 Å wide (Figure 5b and Table S4). All four copies of BCL‐x_L_‐AF7/BCL‐x_L_ in the asymmetric unit have this narrow groove, that is, groove‐closed conformation, thus suggesting that this is independent of crystal packing. For clarity, an overlay of the BCL‐x_L_ domain only, when in complex with BIM, Affimer and WEHI‐539, is presented in Figure S5. This suggests that not only are Affimers selecting a single conformation from the multiple dynamic possibilities in solution, but also that they could be used to select a conformation that is desired by the experimenter. In this case, not only does the Affimer bound conformation correspond to a small molecule bound conformation (thus this might conceptually be a better starting point for structure based drug design), but the groove closed conformation is also a conformation where the groove is too narrow to accommodate the BH3 helix, possibly potentiating the orthosteric competitive effect.

**Figure 6:**
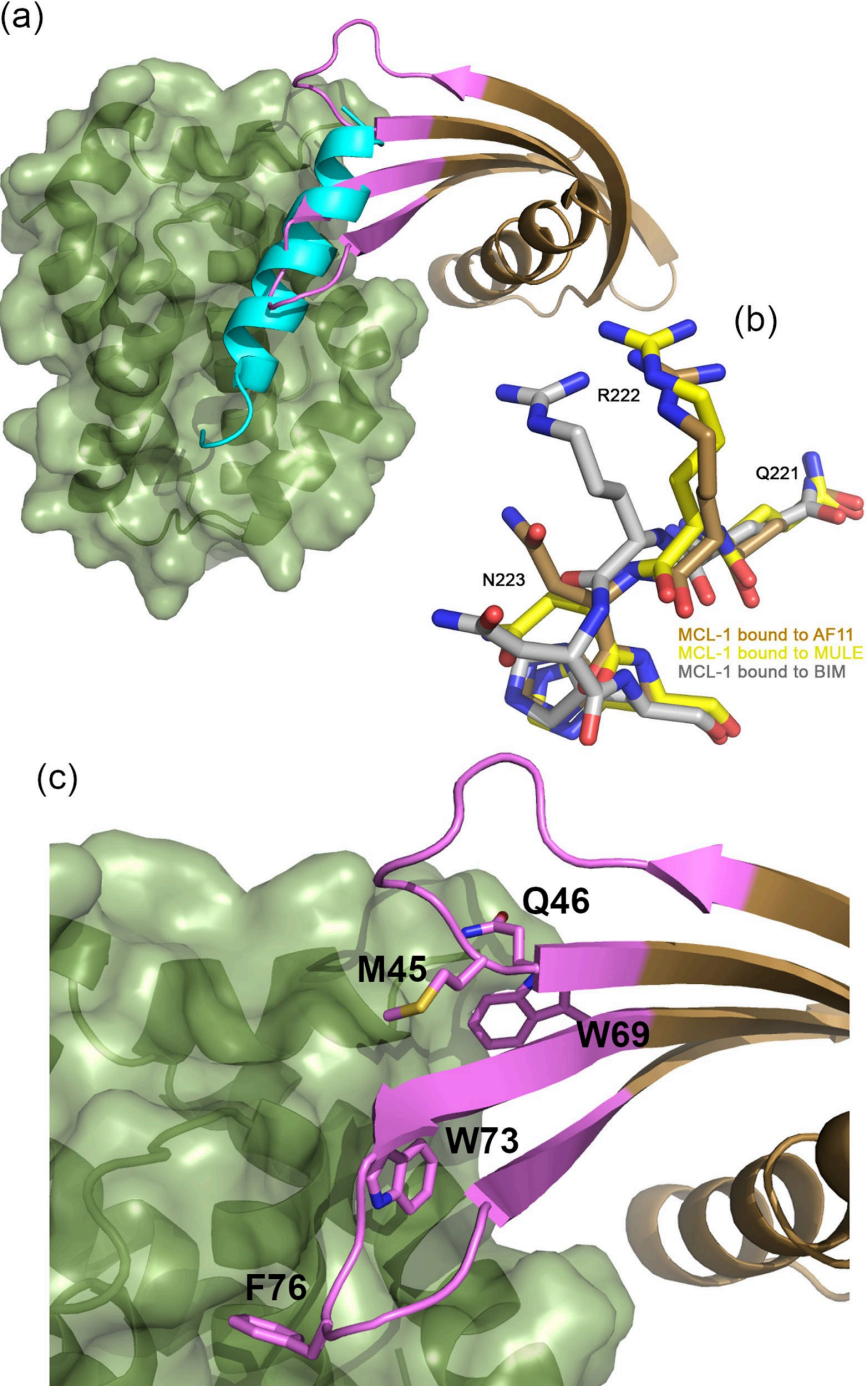
The Affimer/MCL‐1 co‐crystal structure suggests conformational selection. a) Affimer MCL‐1‐AF11 (brown) bound to MCL‐1 (green) illustrating projection of Affimer loops into the BH3 binding cleft. Overlaid on NOXA (cyan)‐bound MCL‐1. b) Ubiquitylatable region of MCL‐1 bound to BIM (grey), Mule (yellow) and MCL‐1‐AF11 (brown). Note the position of the arginine carbonyl group: Mule and Affimer select the desired ubiquitylatable, degradable conformation, whereas BIM does not. c) Enlargement of hydrophobic clusters from VRs 1 and 2 (pink side chains, labelled) projecting into the BH3 cleft.

For MCL‐1‐AF11/MCL‐1, the crystals diffract to 2.20 Å, and the asymmetric unit contains 4 copies of the complex (Figure 6a, b) with representative electron density in Figure S4. Again, the competition with BH3 peptide is mediated by VR residues inserted into the binding groove, with W73 of MCL‐1‐AF11 binding in the same pocket as V85 of NOXA (PDB ID: 2NLA); unsurprisingly, all three crystal structures reveal that the Affimers use the available pockets in the peptide binding site for binding. Again, as for the BCL‐x_L_ Affimers, we observe that the binding of Affimer selects a desirable conformation. Song et al.^[56]^ have shown that MCL‐1 can adopt multiple conformations in solution with differing outcomes in the cell. When BIM BH3 is bound, MCL‐1 adopts a nonhelical conformation at the QRN motif around Arg222. By contrast, when bound to Mule BH3 or a range of small molecules, the QRN motif is helical (Figure 6c). Critically, when the QRN motif is helical, then ubiquitin can be added at this motif, and this promotes cellular degradation by the proteasome. By both competitively inhibiting BH3 binding at the groove, and promoting degradation in cells, the small molecules dramatically reduce MCL‐1 activity in treated cells, promoting apoptosis in MCL‐1 dependent cancer cell lines. Interestingly, our structure shows that MCL‐1‐AF11 also selects the desired helical, ubiquitylatable, conformation (Figure 6c), again demonstrating that an Affimer can be isolated that selects a specific desired conformation. Future studies will focus on exploring the ability of the Affimer to promote this modification.

All known biological partners,^[4]^ and indeed all designed peptides thus far,[^13^, ^14^, ^57^, ^58^, ^59^] that interact with BCL‐2 family proteins at the BH3 groove do so with peptide in a helical conformation. Crucially, the VRs do not adopt an α‐helical conformation to make interactions with the BH3 binding cleft (presumably in part because they are constrained from doing so). Despite the absence of a helical binding conformation, the Affimers project amino acids side chains so as to mimic key hydrophobic and polar contacts made by BH3 ligands and BCL/MCL. Thus, we have identified proteins that bind to the BH3 binding groove via a noncanonical fold. Peptidomimetics based on this structure, rather than the canonical α‐helices, may therefore represent a novel starting point for small molecule discovery.

## Discussion

We have used the Affimer libraries to isolate reagents that are highly selective for their targets and can discriminate between related homologues such as BCL‐2 family proteins. Comparison between the Affimer/BCL‐x_L_ and BCL‐x_L_/BIM (PDB ID: 1PQ1) structures illuminates key features; the BH3 cleft narrows in response to Affimer binding in contrast to the wider cleft observed for binding of BIM (Figure 5a). The BCL‐x_L_ conformation in the Affimer bound form is much more similar to that observed for small molecule bound structures such as WEHI‐539 (PDB ID: 3ZLR. Figure 5b), where BCL‐x_L_ is also domain swapped.

Similarly, when comparing the structures of MCL‐1 bound to peptide versus Affimer we observe that the variable loops of the Affimer are inserted into the BH3 binding groove and that a desirable conformation is selected. In this case, the region where the protein conformation is affected is remote from the BH3 cleft, but again reflects a small molecule bound conformation. For BIM bound MCL‐1, the conformation at QRN (around Arg222) is nonhelical and not ubiquitylatable. In contrast, MCL‐1 bound to small molecules from Song et al.,^[56]^ UMI‐77, Maritoclax and TW‐37, or Affimer MCL‐1‐AF11, have a helical conformation around the QRN motif. The helical QRN motif can be ubiquitylated; BH3 ligands that promote this conformation offer both orthosteric inhibition of BH3 binding and degradation by the proteasome to potentiate the removal of MCL‐1 function in cells. The Affimer again selects a conformation of a target protein to promote a desired functional outcome.

These data, and a previous report,^[34]^ imply that Affimers can be used not only to identify selective sequences that differentiate between related family members, but also that they can be used for conformational selection of productive or desirable binding modes. The role of conformational selection in studies of protein‐protein interactions is increasingly being recognised.[^60^, ^61^, ^62^, ^63^, ^64^, ^65^] Still, it remains a major challenge to account for protein dynamics in structure‐based drug‐design.^[66]^ This library of Affimers allows exploration of a dynamic range of protein target conformers, potentially facilitating generation of template pharmacophores for small‐molecule ligand design and structure based‐ligand design, which may offer an advantage over the current process that typically operates using static crystal structures.^[66]^

## Conclusion

In summary, we have identified unnatural protein ligands that exhibit selectivity for different BCL‐2 family members. Although computational protein design has been applied to discovery of BCL‐2 family selective binders,^[67]^ to our knowledge non‐antibody based binding proteins have not previously been shown to differentiate between these proteins, specifically BCL‐x_L_ and MCL‐1; this is noteworthy given the role of MCL‐1 in driving several cancers.[^68^, ^69^] We note the attrition rate that is a consequence of applying a variety of activity criteria as we progressed along this pipeline. Of the 12 MCL‐1 and 11 BCL‐x_L_ binders identified, not all were inhibitors of the cognate BH3 binding, not all were amenable to biophysical assays, and only three yielded high resolution crystal structures. This serves as a reminder that a large number of binders is required in order to identify ligands with multiple selection criteria applied.

The co‐crystal structures provide inspiration for the structure‐based design of peptide and small molecule based BCL‐2 family modulators. Similarly, BCL‐2 binding Affimers themselves could be elaborated for therapeutic or diagnostic use;[^34^, ^70^] goals we will pursue in due course. Of more generic significance is the observation that noncanonical folds can substitute for native folds in peptide/protein based inhibitors of PPIs;[^71^, ^72^] this is reminiscent of the use of a β‐hairpin to mimic an α‐helix for p53/*h*DM2 inhibition.[^73^, ^74^] In contrast to those studies, the sequences identified here were obtained under selection pressure, and this poses the question: to date BCL‐2 family proteins have only been observed to function in cells through the canonical α‐helix/cleft motif; are there biological modulators of the BCL‐2 family that act through other molecular modes of interaction yet to be discovered?

## Experimental Section

For BCL‐2 family protein expression, biochemical/biophysical screening and details of the crystallography, see the Supporting Information.

**Screening for Affimers** BCL‐2 family proteins were biotinylated by using EZ‐link NHS‐SS‐biotin (Pierce), according to the manufacturer's instructions. Biotinylation was confirmed using streptavidin conjugated to horseradish peroxidase (HRP). Biotin‐BCL‐2 family proteins were added and incubated on pre‐blocked streptavdin plate, the plate was then washed using a KingFisher robotic platform (ThermoFisher) and 10^12^ cfu of the prepanned phage library was added and incubated for 2.5 h with shaking. Wells were washed ten times and eluted with 100 μL 0.2 M glycine (pH 2.2) for 10 min, neutralised with 15 μL 1 M Tris**⋅**HCl (pH 9.1), further eluted with triethylamine 100 mM for 6 min, and neutralised with 1 M Tris**⋅**HCl (pH 7). Eluted phage were used to infect ER2738 cells for 1 h at 37 °C and 90 rpm then plated onto LB agar plates with 100 μg/mL carbenicillin and grown overnight. All colonies were scrapped into 5 mL of 2XYT with carbenicillin (10 μg/mL) and 1×10^9^ M13 K07 helper phage were added. After an overnight incubation phage were precipitated with 4 % poly(ethylene glycol) 8000, 0.3 M NaCl and resuspended in 1 mL of 10 mM Tris, pH 8.0, 1 mM EDTA (TE buffer). 2 μL phage suspension was used for the second round panning round using streptavidin magnetic beads as opposed to streptavidin plates (Invitrogen); otherwise the second pan was conducted in the same way as the first pan. The third pan was conducting using neutravidin high binding capacity plates (Pierce). After the final pan colonies were picked, an ELISA was conducted to select positive clones (in the same way as the enrichment ELISA) which were sent for Sanger sequencing.

**Overexpression and purification of Affimers** The Affimers were subcloned from the phage display vector into pET11a then expressed and purified from *E. coli* strain Rosetta 2. 10 mL of overnight starter culture was used to inoculate 1 L 2×YT containing 125 μg/mL Ampicillin Cultures were grown at 37 °C to an OD_600_ of 0.6–0.8, the temperature was then switched to 18 °C, and protein expression was induced by the addition of 0.5 mM IPTG. Induced cultures were grown at 18 °C overnight before harvesting by centrifugation (Beckman JLS 8.100 rotor, 4 500 rpm, 12 min, 4 °C). Cells were resuspended in 50 mM Tris (pH 8.0), 500 mM NaCl, 15 mM imidazole and lysed by sonication in the presence of 10 μL of 1 U.mL‐1 DNase I per litre of over‐expression culture and cell lysate was clarified (Beckman JA25.50 rotor, 17 000 rpm, 45 min, 4 °C). The supernatant was filtered (0.45 μM syringe filter) before application onto a 5 mL HisTrap that had previously been equilibrated with 50 mM Tris pH 8.0, 500 mM NaCl, 15 mM imidazole. The cleared cell lysate was then allowed to flow through the HisTrap with the aid of a peristaltic pump. The HisTrap was then washed with 10 CV of 50 mM Tris pH 8.0, 500 mM NaCl, 15 mM imidazole followed by 10 CV 50 mM Tris pH 8.0, 500 mM NaCl, 50 mM imidazole and 10 CV 50 mM Tris pH 8.0, 500 mM NaCl, 100 mM imidazole. The Affimer was then eluted from the HisTrap with 50 mM Tris pH 8.0, 500 mM NaCl, 300 mM imidazole. Successful elution was confirmed on a gel before further purification was undertaken. The eluted Affimer was concentrated (Amicon Ultra centrifugal filter, MWCO 10 000) to approximately 5 mL. The sample was then filtered before being loaded onto a Superdex 75 column (GE healthcare) equilibrated in 50 mM Tris (pH 8.0), 250 mM NaCl, 0.5 mM DTT, 2.5 % Glycerol. The protein eluted as a monomer from gel filtration. The purified protein was concentrated to ∼6 mg/mL and stored at −80 °C with the addition of 5 % glycerol.

Additionally, Affimers BCL‐x_L_‐AF6 and MCL‐1‐AF11 were subcloned into pET28a His‐SUMO expression vector to remove flexible residues at the *N* and *C*‐termini which have hindered crystallisation. The constructs were over‐expressed in the *E. coli* strain Rosetta 2. 10 mL of overnight starter culture was used to inoculate 1 L 2 ×YT containing 50 μg/mL kanamycin. Cultures were grown at 37 °C to an OD_600_ of 0.6–0.8, the temperature was then switched to 18 °C, and protein expression was induced by the addition of 0.5 μM IPTG. Induced cultures were grown at 18 °C overnight before harvesting by centrifugation (Beckman JLS 8.100 rotor, 4 500 rpm, 12 min, 4 °C). Cells were re‐suspended in 20 mM Tris pH 8.0, 500 mM NaCl, 15 mM imidazole and lysed by sonication and cell lysate was clarified (Sorvall SS34 rotor, 17 000 rpm, 45 min, 4 °C). The supernatant was filtered (0.45 μM syringe filter) before application onto a 5 mL HisTrap that had previously been equilibrated with 20 mM Tris pH 8.0, 500 mM NaCl, 15 mM imidazole. The HisTrap was then washed with 10 CV of 50 mM Tris pH 8.0, 500 mM NaCl, 15 mM imidazole followed by 10 CV 50 mM Tris pH 8.0, 500 mM NaCl, 50 mM imidazole. The His‐SUMO‐Affimer fusion protein was then eluted from the HisTrap with 20 mM Tris pH 8.0, 500 mM NaCl, 300 mM imidazole. The His‐SUMO‐Affimer fusion protein was cleaved overnight in dialysis into 20 mM Tris pH 8.0, 250 mM NaCl in the presence of Smt3 protease, Ulp1, overnight at 4 °C. To remove any uncleaved Affimer, His‐SUMO and Ulp1, the sample was reapplied to a HisTrap in 20 mM Tris pH 8.0, 250 mM NaCl and the flow through containing Affimer collected. This was concentrated (Amicon Ultra centrifugal filter, MWCO 10 000) to approximately 10 mL. The sample was then filtered before being loaded onto a Superdex 75 column (GE healthcare) equilibrated in 20 mM Tris (pH 8.0), 250 mM NaCl, 0.5 mM DTT, 2.5 % glycerol. The protein eluted as a monomer from gel filtration. The purified protein was concentrated to ∼6 mg/mL and stored at −80 °C with the addition of 5 % glycerol.

## Author Contributions

All authors have read and approved the manuscript. F.H. and J.A.M. performed experiments, analysed data and wrote the manuscript. J.T., P.R.R., P.R., H.F.K., C.T., C.H.T., B.J., F.N. and B.I.M.W. performed experiments and analysed data. D.C.T. and J.C. designed experiments and applied for funding. A.J.W. and T.A.E. applied for funding, designed and performed experiments, analysed data and wrote the manuscript.

## Conflict of interest

The authors declare no conflict of interest.

## Supporting information

As a service to our authors and readers, this journal provides supporting information supplied by the authors. Such materials are peer reviewed and may be re‐organized for online delivery, but are not copy‐edited or typeset. Technical support issues arising from supporting information (other than missing files) should be addressed to the authors.

Supplementary
