# Supplementary material for: Selective Affimers Recognise the BCL‐2 Family Proteins BCL‐xL and MCL‐1 through Noncanonical Structural Motifs
Source: Chembiochem. 2020 Nov 2;22(1):232–40. doi: 10.1002/cbic.202000585 (PMC7821230; doi:10.1002/cbic.202000585)
Supplement: Supplementary file 1 — Supplementary [file CBIC-22-232-s001.pdf]

ChemBioChem

Supporting Information

Selective Affimers Recognise the BCL-2 Family Proteins BCL-x_L and MCL-1 through Noncanonical Structural Motifs**

Jennifer A. Miles[+], Fruzsina Hobor[+], Chi H. Trinh, James Taylor, Christian Tiede, Philip R. Rowell, Brian R. Jackson, Fatima A. Nadat, Pallavi Ramsahye, Hannah F. Kyle, Basile I. M. Wicky, Jane Clarke, Darren C. Tomlinson, Andrew J. Wilson,* and Thomas A. Edwards*

Author Contributions

J.M. Formal analysis:Equal; Investigation:Equal; Methodology:Equal; Validation:Equal; Writing – original draft:Equal; Writing – review & editing:Equal
F.H. Investigation:Equal; Methodology:Equal; Validation:Equal; Writing – original draft:Equal; Writing – review & editing:Equal
c.t. Data curation:Equal; Investigation:Equal; Validation:Equal
J.T. Investigation:Equal
C.T. Investigation:Equal; Methodology:Equal
P.R. Investigation:Equal
B.J. Investigation:Equal; Methodology:Equal
F.N. Investigation:Equal
P.R. Investigation:Equal
H.K. Investigation:Equal
B.W. Investigation:Equal
J.C. Supervision:Equal
D.T. Supervision:Equal
A.W. Conceptualization:Equal; Funding acquisition:Equal; Investigation:Equal; Methodology:Equal; Project administration:Equal; Supervision:Equal; Validation:Equal; Writing – original draft:Equal; Writing – review & editing:Equal
t.e. Conceptualization:Equal; Data curation:Equal; Funding acquisition:Equal; Investigation:Equal; Project administration:Equal; Supervision:Equal; Validation:Equal; Writing – original draft:Equal; Writing – review & editing:Equal

Additional Materials and Methods

BCL-x_L and MCL-1 expression

BCL-x_L and MCL-1 were overexpressed and purified according to our published procedure.[1] The pet28a His-SUMO Mcl-1 (172-327) and pet28a His-SUMO Bcl-x_L (a chimera with BCL-2, 1-198, missing 27-82)[2, 3] constructs were over-expressed in the *E.coli* strain Rosetta 2. 10 ml of overnight starter culture was used to inoculate 1 L 2 xYT containing 50 µg/ml Kanamycin. Cultures were grown at 37 °C until OD600 ~ 0.6 – 0.8, the temperature was then switched to 18 °C and protein expression induced by the addition of 0.5uM IPTG. Induced cultures were grown at 18 °C overnight before harvesting by centrifugation (Beckman JLS 8.100 rotor, 4,500 rpm, 12 min, 4 °C). Cells were resuspended in 20mM TRIS pH 8.0, 500mM NaCl, 15mM Imidazole and lysed by sonication and cell lysate was clarified (Sorvall SS34 rotor, 17,000 rpm, 45 min, 4 °C). The clarified lysate was applied to a 5ml HisTrap equilibrated with 20mM TRIS pH 8.0, 500mM NaCl, 15mM Imidazole. The HisTrap was then washed with 10 CV of 20mM TRIS pH 8.0, 500mM NaCl, 15mM Imidazole followed by 10 CV 20mM TRIS pH 8.0, 500mM NaCl, 25mM Imidazole. The fusion proteins were eluted in 20mM TRIS pH 8.0, 500mM NaCl, 300mM Imidazole. The His-SUMO tag was removed in overnight in dialysis into 20mM TRIS pH 8.0, 250mM NaCl in the presence of Smt3 protease, Ulp1, overnight at 4°C. Uncleaved material and His-SUMO were removed by reapplication of the sample to a HisTrap in 20mM TRIS pH 8.0, 250mM NaCl and the flow through containing Mcl-1 or Bcl-xl collected. The proteins were then filtered before further purification on a Superdex 75 (GE healthcare) equilibrated in 20mM TRIS pH 8.0, 250mM NaCl, 0.5mM DTT, 2.5% Glycerol. Purified proteins were concentrated and stored at -80°C.

BCL-2 expression

Glutathione S-transferase (GST) tagged BCL-$2^{1\text{-}205}$ fusion protein constructs were over-expressed in *E.coli* BL21 (DE3) Rosetta 2, and purified. 10 ml of overnight starter culture was used to inoculate 1 L 2 xYT containing 100 µg/ml Ampicillin. Cultures were grown at 37 °C until OD_{600} reached 0.6 – 0.8. The temperature was reduced to 18 °C

and protein expression induced by adding 0.3 mM IPTG. Induced cultures were grown at 18°C overnight before harvesting by centrifugation (Beckmann JLS 8.1, 5000rpm, 15 minutes, 4°C). Cell pellets were re-suspended in lysis buffer (50mM TRIS pH 8.0, 200mM NaCl, 5mM DTT, 1mM EDTA) and lysed by sonication in the presence of 10 µL of 1 U.ml-1 DNase I per litre of over-expression culture. Cell lysate was clarified (Sorvall SS34 rotor, 18,000 rpm, 45 min, 4°C), and the supernatant was filtered (0.45 µM Minisart, Sartorius). Clarified cell lysate was added to approximately 10 mL Glutathione Superflow Resin (Generon) packed in a free-flow gravity column. The column was washed with 5 column volumes of water and equilibrated with 5 column volumes of lysis buffer. The lysate was then added to the column and placed on an analogue roller mixer (SKS science) at 4°C for 1-3 hours. Lysate was then eluted from the column under gravity, and the resin was washed first with 100 mL high salt wash buffer (50 mM Tris pH 8, 1 mM DTT, 1 mM EDTA, 500 mM NaCl, 10% glycerol, 0.01% Triton), then 100 mL low salt wash buffer (50 mM Tris pH 8, 1 mM DTT, 1 mM EDTA, 200 mM NaCl, 10% glycerol, 0.01% Triton). The resin was re-suspended in 20 mL of low salt wash buffer, supplemented with 400 µL PreScission protease to cleave the GST tag from the fusion protein. Following overnight incubation on an analogue roller mixer (SKS science) at 4°C, cleaved protein was obtained by collecting the flow through from the column. To collect all cleaved protein, the resin was washed with 50 mL of low salt wash buffer, and the flow through collected. All wash fractions were collected and analysed by SDS-PAGE. Cleaved protein was concentrated (Amicon, MWCO 10,000) to 5 ml, filtered (0.22 µM Minisart, Sartorius) and further purified by Size Exclusion Chromatography. Purified protein was concentrated and stored at -80°C, with the addition of 5% glycerol to aid long term stability.

BAK and BAX expression

Mxe intein / chitin binding domain (CBD) tagged fusion protein constructs were over-expressed in *E.coli* C41 (DE3) cells, and purified. 2 mL of overnight starter culture was used to inoculate 1 L LB containing 100 µg/mL Ampicillin. Cultures were grown at 37°C until OD_{600} reached 0.6 – 0.8. For $BAX^{1\text{-}171(C62S\ C126S)}$ protein expression was induced by adding 0.1 mM IPTG. Induced cultures were grown at 28°C overnight. For $BAK^{16\text{-}185(C166S)}$ protein expression was induced by adding 1 mM IPTG, and induced cultures were grown at 37°C for 4 hours. Cells were harvested by centrifugation (Beckmann JLS 8.1, 5000rpm, 15minutes, 8°C). Cell pellets were resuspended in lysis buffer (20

mM HEPES pH7.0, 100 mM NaCl, 1 mM EDTA)) and lysed by sonication. Cell lysate was clarified (Sorvall SS34 rotor, 18,000 rpm, 45 min, 8°C), the supernatant was filtered (0.22 μM Minisart, Sartorius). Clarified cell lysate was added to approximately 20 mL Chitin Resin (New England Biolabs) packed in a free-flow gravity column, equilibriated with 20 column volumes of lysis buffer. The lysate was then added to the column and eluted from the column at a flow rate of 1 mL/min, then the resin was washed with 10 column volumes of lysis buffer. The column was quickly equilibrated with 3 column volumes of lysis buffer supplemented with 50 mM DTT. The resin was then resuspended in 1 column volume of lysis buffer supplemented with 50 mM DTT. Following overnight incubation on an analogue roller mixer (SKS science) at 25°C, cleaved protein was obtained by collecting the flow through from the column. To collect all cleaved protein, the resin was washed with 50 mL of lysis buffer, then a further 100 mL of lysis buffer. All wash fractions were collected and analysed by SDS-PAGE. Columns were stored in 20% ethanol. Cleaved protein was concentrated (Amicon, MWCO 10,000) to 10 mL, filtered (0.22 μM Minisart, Sartorius) and further purified by Size Exclusion Chromatography. Purified protein was stored at 4°C without further concentration.

Screening for Affimers

BCL-2 family proteins were biotinylated using EZ-link NHS-SS-biotin (Pierce), according to the manufacturer's instructions. Biotinylation was confirmed using streptavidin conjugated to horseradish peroxidase (HRP). Biotin-BCL-2 family proteins were added and incubated on pre-blocked steptavdin plate, the plate was then washed using a KingFisher robotic platform (ThermoFisher) and 10^{12} cfu of the prepanned phage library was added and incubated for 2.5 h with shaking. Wells were wash ten times and eluted with 100 μL 0.2 M glycine (pH 2.2) for ten minutes neutralized with 15 μL 1 M Tris-HCl (pH 9.1), further eluted with triethylamine 100 mM for 6 min, and neutralised with 1 M Tris–HCl (pH 7). Eluted phage were used to infect ER2738 cells for 1 h at 37 °C and 90 rpm then plated onto LB agar plates with 100 μg/ml carbenicillin and grown overnight. All colonies were scrapped into 5 mL of 2XYT with carbenicillin (10 μg/mL) and 1 x 10^9 M13K07 helper phage were added. After an overnight incubation phage were precipitated with 4 % polyethylene glycol 8000, 0.3 M NaCl and resuspended in 1 ml of 10 mM Tris, pH 8.0, 1 mM EDTA (TE buffer). 2 μL phage suspension was used for the second round panning round using streptavidin magnetic beads as opposed to streptavidin plates (Invitrogen); otherwise the second pan was conducted in the same way as the first pan. The third pan was conducting using neutravidin high binding capacity plates

(Pierce). After the final pan colonies were picked, an ELISA was conducted to select positive clones (in the same way as the enrichment ELISA) which were sent for Sanger sequencing.

Overexpression and purification of Affimers

The Affimers were subcloned from the phage display vector into pET11a then expressed and purified from *E. coli* strain Rosetta 2. 10 ml of overnight starter culture was used to inoculate 1 L 2 x YT containing 125 µg/ml Ampicillin Cultures were grown at 37 °C until OD600 ~ 0.6 – 0.8, the temperature was then switched to 18 °C and protein expression induced by the addition of 0.5mM IPTG. Induced cultures were grown at 18 °C overnight before harvesting by centrifugation (Beckman JLS 8.100 rotor, 4,500 rpm, 12 min, 4 °C). Cells were resuspended in 50 mM TRIS pH 8.0, 500 mM NaCl, 15 mM imidazole and lysed by sonication in the presence of 10 µL of 1 U.ml-1 DNase I per litre of over-expression culture and cell lysate was clarified (Beckman JA25.50 rotor, 17,000 rpm, 45 min, 4 °C). The supernatant was filtered (0.45 µM syringe filter) before application onto a 5 ml HisTrap that had previously been equilibrated with 50 mM TRIS pH 8.0, 500 mM NaCl, 15 mM imidazole. The cleared cell lysate was then allowed to flow through the HisTrap with the aid of a peristaltic pump. The HisTrap was then washed with 10 CV of 50 mM TRIS pH 8.0, 500 mM NaCl, 15 mM imidazole followed by 10 CV 50 mM TRIS pH 8.0, 500 mM NaCl, 50 mM imidazole and 10 CV 50 mM TRIS pH 8.0, 500 mM NaCl, 100 mM imidazole. The Affimer was then eluted from the HisTrap with 50 mM TRIS pH 8.0, 500 mM NaCl, 300 mM imidazole. Successful elution was confirmed on a gel before further purification was undertaken. The eluted Affimer was concentrated (Amicon Ultra centrifugal filter, MWCO 10,000) to approximately 5 ml. The sample was then filtered before being loaded onto a Superdex 75 column (GE healthcare) equilibrated in 50 mM TRIS pH 8.0, 250 mM NaCl, 0.5 mM DTT, 2.5% Glycerol. The protein eluted as a monomer from gel filtration. The purified protein was concentrated to ~ 6 mg/ml and stored at – 80 °C with the addition of 5% Glycerol.

Additionally, Affimers **BCL-x$_L$-AF6** and **MCL-1-AF11** were subcloned into pET28a His-SUMO expression vector to remove flexible residues at the *N* and *C*-termini which have hindered crystallisation. The constructs were over-expressed in the *E. coli* strain Rosetta 2. 10 ml of overnight starter culture was used to inoculate 1 L 2 xYT containing 50 µg/ml Kanamycin. Cultures were grown at 37 °C until OD600 ~ 0.6 – 0.8, the temperature was then switched to 18 °C and protein expression induced by the addition of 0.5uM IPTG. Induced cultures were grown at 18 °C overnight before harvesting by centrifugation (Beckman JLS 8.100 rotor, 4,500 rpm, 12 min, 4 °C). Cells were re-suspended in 20 mM TRIS pH 8.0, 500 mM NaCl, 15 mM imidazole and lysed by sonication and cell lysate was clarified (Sorvall SS34 rotor, 17,000 rpm, 45 min, 4 °C). The supernatant was filtered (0.45 µM syringe filter) before application

onto a 5 ml HisTrap that had previously been equilibrated with 20 mM TRIS pH 8.0, 500 mM NaCl, 15 mM imidazole. The HisTrap was then washed with 10 CV of 50 mM TRIS pH 8.0, 500 mM NaCl, 15 mM imidazole followed by 10 CV 50 mM TRIS pH 8.0, 500 mM NaCl, 50 mM imidazole. The His-SUMO-Affimer fusion protein was then eluted from the HisTrap with 20 mM TRIS pH 8.0, 500 mM NaCl, 300 mM imidazole. The His-SUMO-Affimer fusion protein was cleaved overnight in dialysis into 20 mM TRIS pH 8.0, 250 mM NaCl in the presence of Smt3 protease, Ulp1, overnight at 4 °C. To remove any uncleaved Affimer, His-SUMO and Ulp1, the sample was reapplied to a HisTrap in 20 mM TRIS pH 8.0, 250 mM NaCl and the flow through containing Affimer collected. This was concentrated (Amicon Ultra centrifugal filter, MWCO 10,000) to approximately 10 ml. The sample was then filtered before being loaded onto a Superdex 75 column (GE healthcare) equilibrated in 20 mM TRIS pH 8.0, 250 mM NaCl, 0.5 mM DTT, 2.5% Glycerol. The protein eluted as a monomer from gel filtration. The purified protein was concentrated to ~ 6 mg/ml and stored at – 80 °C with the addition of 5% Glycerol.

Isothermal Titration Calorimetry

Kinetic information of the Affimer interactions with the Bcl-2 family proteins was established using the ITC200 microcalorimeter (MicroCal, Northampton, MA). Sample buffers were 50 mm Tris, pH 7.9, 200 mM NaCl. Experiments were carried out at 30 °C. The syringe contained 40 µl of 350 µM Affimer (**BCL-x$_L$-AF6**, **BCL-x$_L$-AF7** or **MCL-1-AF11**); 2 µl injections were applied every 180 seconds. The cell contained 205 µl of 35 µM BCL-x$_L$ or MCL-1. Cell concentrations were adjusted to a 1:1 stoichiometric interaction and Microcal Origin software version 7.0 was used to determine the dissociation constants (K_d). All measurements were repeated at least twice.

Single Point Fluorescence Anisotropy

A single point assay was carried out at a fixed concentration of Affimer (1 µM), FITC-Ahx-*m*NOXA-B$_{68-87}$ or BODIPY-Ahx-BAK$_{72-87}$ at 87.5nM or 37.5nM respectively and MCL-1 or BCL-x$_L$ at 175 nM or 50nM respectively in phosphate buffer (40 mM sodium phosphate, 200 mM sodium chloride, 0.02 mg ml^{-1} Bovine serum albumin, pH 7.50). Each Affimer was assessed in triplicate and left to equilibrate for 45 minutes in the dark. A positive control was present on each test plate (BAK$_{72-87}$ for the BCL-x$_L$/BAK interaction and mNOXA-B$_{68-87}$ for the MCL-1/NOXA-B interaction) at the same concentration as the test compounds. Anisotropy values were then determined and a percentage of efficiency was calculated for each compound relative to BAK or mNOXA-B's efficiency, with blank wells set to zero.

Competition assays

Competition fluorescence anisotropy assays and data processing were performed adapting previously described protocols.[1] Briefly, the buffer used for fluorescence anisotropy was phosphate buffer (40 mM sodium phosphate, 200 mM sodium chloride, 0.02 mg ml^{-1} Bovine serum albumin, pH 7.50). Assays were run in triplicate in 384 well Optiplates and were scanned using a Perkin Elmer EnVisionTM 2103 MultiLabel plate reader. Fluorescein labelled peptides used an excitation and emission wavelength of 490 nm and 535 nm respectively whilst BODIPY labelled peptides used an excitation and emission wavelength of 531 nm and 595 nm respectively, with a bandwidth of 5 nm. BODIPY-Ahx-BAK_{72-87} /BCL-x_L competition assays were performed in 384 well plates in phosphate buffer with the concentration of the inhibitor typically starting from 5-50 µM, diluted over 24 points in a 2/3 regime with [BODIPY-Ahx-BAK_{72-87}] and [BCL-x_L] fixed at 50 nM and 150 nM respectively. Plates were read after 45 minutes of incubation. FITC-Ahx-*m*NOXA-B_{68-87}/MCL-1 competition assays were performed in a 384 well plates in phosphate buffer, with the concentration of the inhibitor typically starting from 5-100 µM, diluted over 24 points in a 2/3 regime and with [FITC-Ahx-*m*NOXA-B_{68-87}] and [MCL-1] fixed at 50 nM and 150 nM respectively. Plates were read after 45 minutes.

Co-crystallisation

BCL-x_L was incubated with an excess of BCL-x_L-AF7 overnight, before co-purification in 200mM NaCl, 50mM TRIS pH 8.0, 0.5mM DTT via gel filtration on a Superdex75 column. Crystals grew in 12% PEG 1500, 0.1M Sodium Acetate pH 5.5, 2.5 M NaCl, 1.5% MPD at 20°C at 5mg/ml using the sitting drop vapour diffusion method. The crystals were cryoprotected in 20% glycerol and data collected at the Diamond Light Source on beamline i04-1 to 2.24 Å resolution at 100K. The diffraction images were integrated, scaled and reduced using the suite of program XIA2[4] with five percent of the reflections selected at random and excluded from the refinement using FREERFLAG.[5] The unit cell parameters for the crystal are a=68.3Å, b=87.3Å, c=112.2Å, α=90.0°, β=96.2°, γ=90.0° in space group $P2_1$ with four BCL-x_L-AF7/BCL-$_{xL}$ complexes in the asymmetric unit cell. The data processing statistics are shown in Table 2. The structure was determined by molecular replacement using the program PHASER[6] with the human BCL-$_{xL}$ structure (PDB code 1R2D),[7]) and the truncated Affimer (PDB code 4N6T,[8]) as the search models. Manual inspection of electron density maps with iterative cycles of model building and refinement were carried out using COOT[9] and REFMAC5.[10, 11] During the course of model building structural validations were carried out using the program MOLPROBITY.[12] All refinement statistics are shown in Table 2. The structures have been deposited in the Protein Data Bank (www.pdb.org) and has been assigned the PDB codes 6ST2 (BCL-x_L-AF6/Bcl-$_{xL}$) 6HJL (BCL-x_L-AF7/Bcl-$_{xL}$) and 6STJ MCL-1-AF11/MCL-1). Structures were analysed and figures prepared with PyMol[13].

BCL-x_L was incubated with an excess of BCL-xL-AF6 at room temperature for 3 hours. The complex was purified via gel filtration on a Superdex 200 column equilibrated with 20 mM Tris pH 7.4, 50 mM NaCl, 2 mM DTT. Crystals grew in 0.1 M Tris pH 7, 0.2 M MgCl2, 10% w/v PEG 8K at 20°C at 10 mg ml-1 concentraton using the sitting drop vapour diffusion method. The crystals were cryoprotected with 20% glycerol and data was collected at the Diamond Light Source beamline i03 to 1.90 Å resolution at 100K. The unit cell parameters for the crystal are a=71.9Å, b=71.9Å, c=204.2Å, α=90.0°, β=90°, γ=90.0° in space group P41 2 2 with two BclXL:BCL-xL-AF6 complexes in the asymmetric unit cell. The structure was solved using similar strategy as detailed above for BclXL:BCL-xL-AF7 complex.
Mcl-1 was incubated with an excess of MCL-1-AF11 at room temperature for 3 hours. The complex was purified via gel filtration on a Superdex 200 column equilibrated with 20 mM Tris pH 7.4, 50 mM NaCl, 2 mM DTT. Crystals grew in 0.1 M Na Acetate pH 4.6, 30 % w/v PEG MME 2K, 0.2 M (NH4)2SO4 at 20°C at 9 mg ml-1 concentration using the sitting drop vapour diffusion method. The crystals were cryoprotected with 20% glycerol and data was collected at the Diamond Light Source beamline i04-1 to 2.2Å resolution at 100K. The unit cell parameters for the crystal are a=92.1Å, b=107.5Å, c=226.2Å, α=90.0°, β=90°, γ=90.0° in space group C 2 2 21 with four Mcl-1:Mcl-1-AF11 complexes in the asymmetric unit cell. The structure was determined by molecular replacement using the program PHASER[6] with the human Mcl1 structure (PDB code 5FC4),[7]) as the search model. Model building, refinement and structural validations were carried out using the strategy similar to that for the BclX: affimer complexes already described.

All the structures have been deposited in the Protein Data Bank (www.pdb.org) and has been assigned the PDB codes 6ST2 (AF6:Bcl-$_{xL}$) 6HJL (AF7:Bcl-$_{xL}$) and 6STJ AF11:MCL-1). Structures were analysed and figures prepared with PyMol[13].

Additional Data and Figures

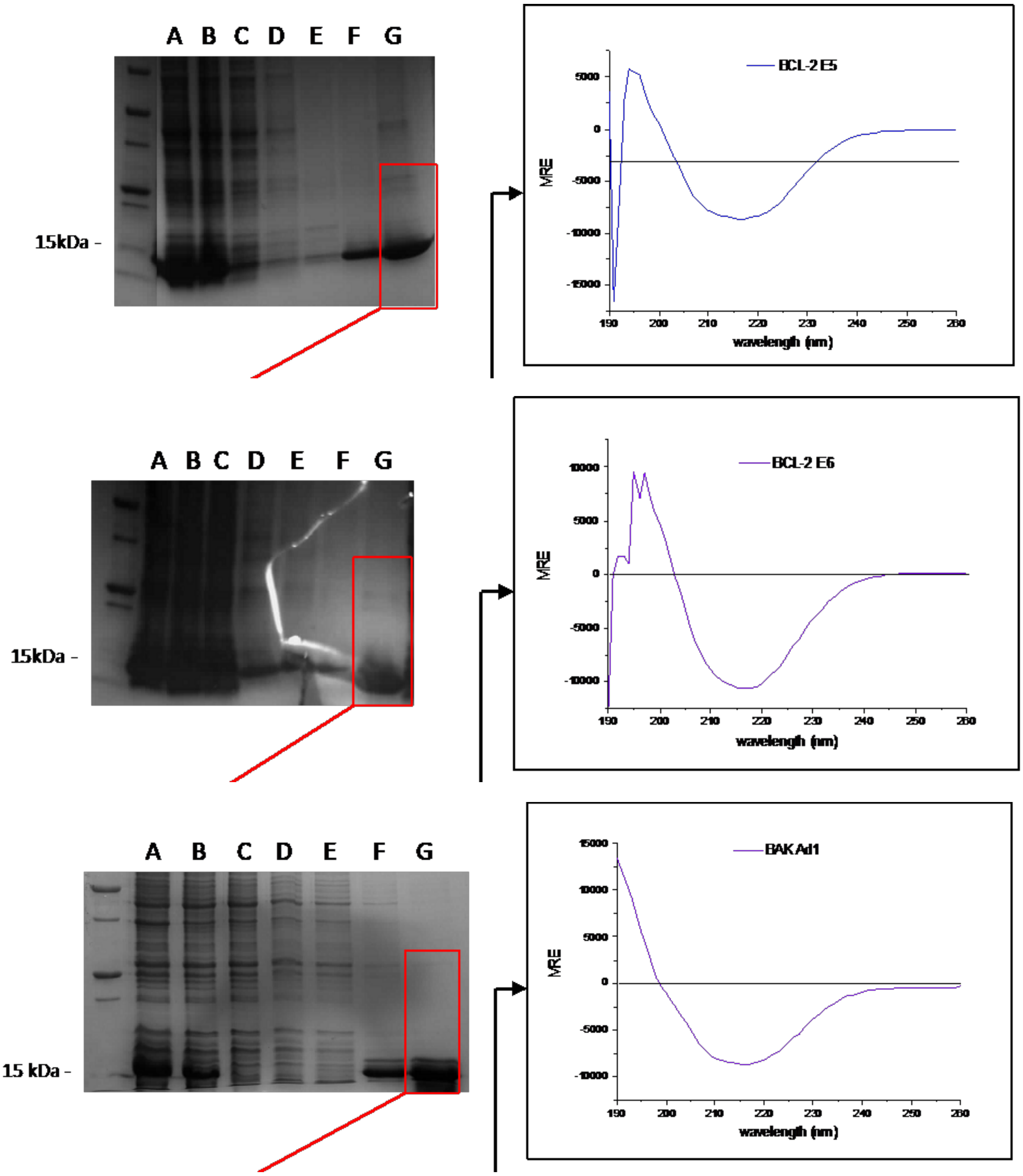

Figure S1. Purification and representative CD analyses for BCL-2 and BAK binding Affimers

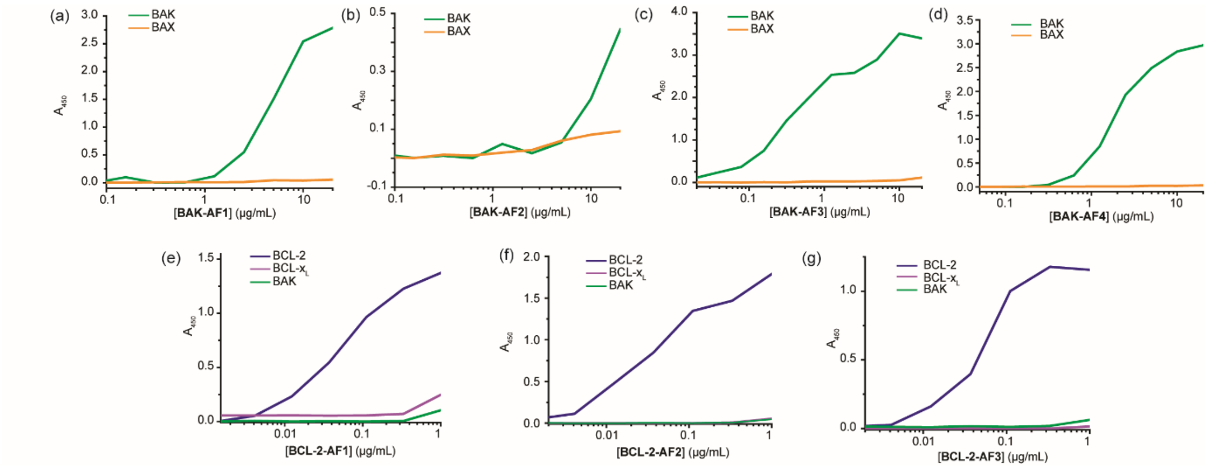

Figure S2. Binding analyses of BCL-2 family binding Affimers; binding ELISA for (a) **BAK-AF1**, (b) **BAK-AF2**, (c) **BAK-AF3**, (d) **BAK-AF4**, (e) **BCL-2-AF3**, (f) **BCL-2-AF3**, (g) **BCL-2-AF3**.

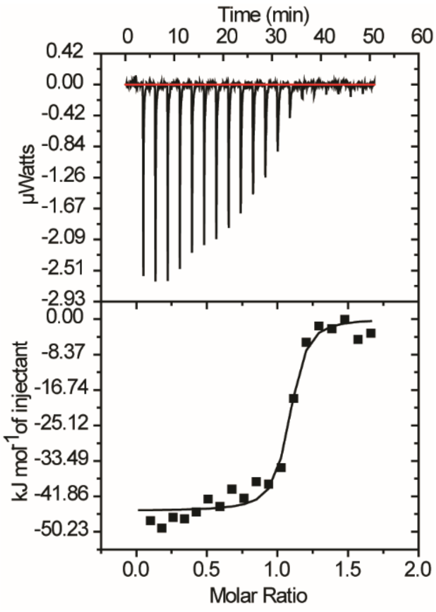

Figure S3. ITC data for Binding of **BCL-x$_L$-AF6** to BCL-x$_L$

Table S1. sequence information and frequency for Affimers selected against BCL-2 Family

Protein	Ligand	VR1	VR2	Frequency
MCL-1	**MCL-1-AF1**	T T P E P Y E Q Q	W W Q G F Q G M V	5
MCL-1	**MCL-1-AF2**	W D Y M G Y S D D	W W Y G F T G W Q	2
MCL-1	**MCL-1-AF3**	S R E N V E S W A	F W Q G F F S I M	7
MCL-1	**MCL-1-AF4**	F S D T P A Q D S	E W M G F F S M A	
MCL-1	**MCL-1-AF5**	K A Q K E V V A E	F W Q G F Y N W V	2
MCL-1	**MCL-1-AF6**	K S A Y D G A W D	N W M G F Y N W D	
MCL-1	**MCL-1-AF7**	W R M Q Y R I G W	P V Y F S N P A M	
MCL-1	**MCL-1-AF8**	V F P S Q D P Q Q	Y W M G F I S W A	
MCL-1	**MCL-1-AF9**	T P F A G D Q M Q	T W K G F F K N D	
MCL-1	**MCL-1-AF10**	F P W M D W L D Q	W W Q G F F Q V E	
MCL-1	**MCL-1-AF11**	M G V N P E E M Q	W W W G F H I W D	
MCL-1	**MCL-1-AF12**	S R E N V E S W A	F W Q G F F S I M	
BCL-xL	**BCL-xL-AF1**	Q F G M A W Y H S	E F V N Q P W S S	2
BCL-xL	**BCL-xL-AF2**	H A R D D C M V L	T F H I A G Y I S	6
BCL-xL	**BCL-xL-AF3**	Q T Q L W L S L V	N L D Q R R G L M	6
BCL-xL	**BCL-xL-AF4**	S V Y M G I S V L	L L K V L D I W H	2
BCL-xL	**BCL-xL-AF5**	Y E I G Y T K N S	V L W N V I Y K V	
BCL-xL	**BCL-xL-AF6**	E R N S I F E E F	R D L V F G G P E	
BCL-xL	**BCL-xL-AF7**	M F S W L D W E E	P A L L W S P H G	
BCL-xL	**BCL-xL-AF8**	E Q R L W F S N V	L S G A R R G I Y	
BCL-xL	**BCL-xL-AF9**	M T Q Y D A Y R N	D I P G L F V N L	
BCL-xL	**BCL-xL-AF10**	Q H H W F E F V D	Q D F F D Q Y R P	
BCL-xL	**BCL-xL-AF11**	S Y E V W A F R Q	R D D Y F W L P K	
BCL-2	**BCL-2-AF1**	S A F S W W E R I	D H S Q P I Q M R	9
BCL-2	**BCL-2-AF2**	G I G F W V A F S	S S K P N Q M M M	
BCL-2	**BCL-2-AF3**	F M E N N G F V M	K W F F L P P I N	9
BCL-2	**BCL-2-AF4**	K M H N S H I I M	K W Y L F P I G A	

Table S2. sequence information and frequency for Affimers selected against BCL-2 Family

Protein	Ligand	VR1	VR2	Frequency
BAX	**BAX-AF1**	H V Q A H F W S I	P T E N I M G L K	22
BAX	**BAX-AF2**	M Q L S S T R L W	T K Y T I Y N P I	
BAX	**BAX-AF3**	E E A V P W Y M P	N Y I K L K W H H	
BAX	**BAX-AF4**	Q V H M H W Y H A	H L K Q H P L I K	
BAX	**BAX-AF5**	N Q A P T F F Q Y	E T K W S H F I Q	
BAK	**BAK-AF1**	V G R H S D W A P	Y P I Q S H P P W	11
BAK	**BAK-AF2**	F V F P H D Y F P	E I A Q W N G W F	2
BAK	**BAK-AF3**	I M A R P P F E I	A Q L K K T L Y W	8
BAK	**BAK-AF4**	I W N N S H Y I P	Y P L S L K P F I	

Table S3. Data collection, processing and refinement statistics for Affimer:target complexes

	AF6:Bcl-$_{xL}$	AF7:Bcl-$_{xL}$	AF11:MCL-1
Resolution range (Å) *	102.1-1.79 (1.84-1.79)	40.39–2.24 (2.30-2.24)	113.1-2.20 (2.24-2.20)
Space group	$P\,4_1\,2\,2$	$P\,2_1$	$C\,2\,2\,2_1$
Unit-cell parameters. a, b, c (Å), α β γ °	71.9, 71.9, 204.2, 90, 90, 90	68.3, 87.3, 112.2, 90, 96.2, 90	92.14, 107.5, 226.2, 90, 90, 90
No. of observed reflections		202,533	
No. of unique reflections	51,639	61,813	57,158
Redundancy	13.9 (14.2)	3.3 (3.3)	
Completeness (%) *	100.0 (100.0)	98.1 (97.8)	99.4 (91.4)
< $I/\sigma(I)$ >*	13.8 (1.1)	12.6 (1.4)	13.5 (1.2)
R_{merge} §*	0.079 (4.694)	0.041 (0.830)	0.083 (1.644)
R_{pim} ¥*	0.031 (1.835)	0.039 (0.780)	0.025 (0.591)
$CC_{1/2}$	0.991 (0.533)	0.72	0.988 (0.681)
R factor (%)	21.4	23.8	25.7
R_{free} (%)†	24.1	28.6	29.0
No. of protein non-H atoms	3,762	7,402	7,253
No. of water molecules	94	0	51
R.m.s.d bond lengths (Å) ξ	0.007	0.014	0.005
R.m.s.d bond angles (˚) ξ	1.4	1.7	1.3
Average overall B factor (Å^2)			
Protein	50	66	66
Residues in the regions of Ramachandran plot (%) ‡			
Favoured region	98.7	91.0	96.3
Outliers	0	2.7	0.2
PDB code	6ST2	6HJL	6STJ

*Values given in parentheses correspond to those in the outermost shell of the resolution range.

§ $R_{merge} = \sum_{hkl}\sum_i \left|I_i(hkl) - \langle I(hkl)\rangle\right| / \sum_{hkl}\sum I_i(hkl)$

¥ $R_{pim} = \sum_{hkl}\{1/[N(hkl)-1]\}^{1/2}\sum_i \left|I_i(hkl) - \langle I(hkl)\rangle\right| / \sum_{hkl}\sum_i I_i(hkl)$

† R_{free} was calculated with 5% of the reflections set aside randomly.

ξ Based on the ideal geometry values of Engh and Huber.

‡ Ramachandran analysis using the program MolProbity.[10]

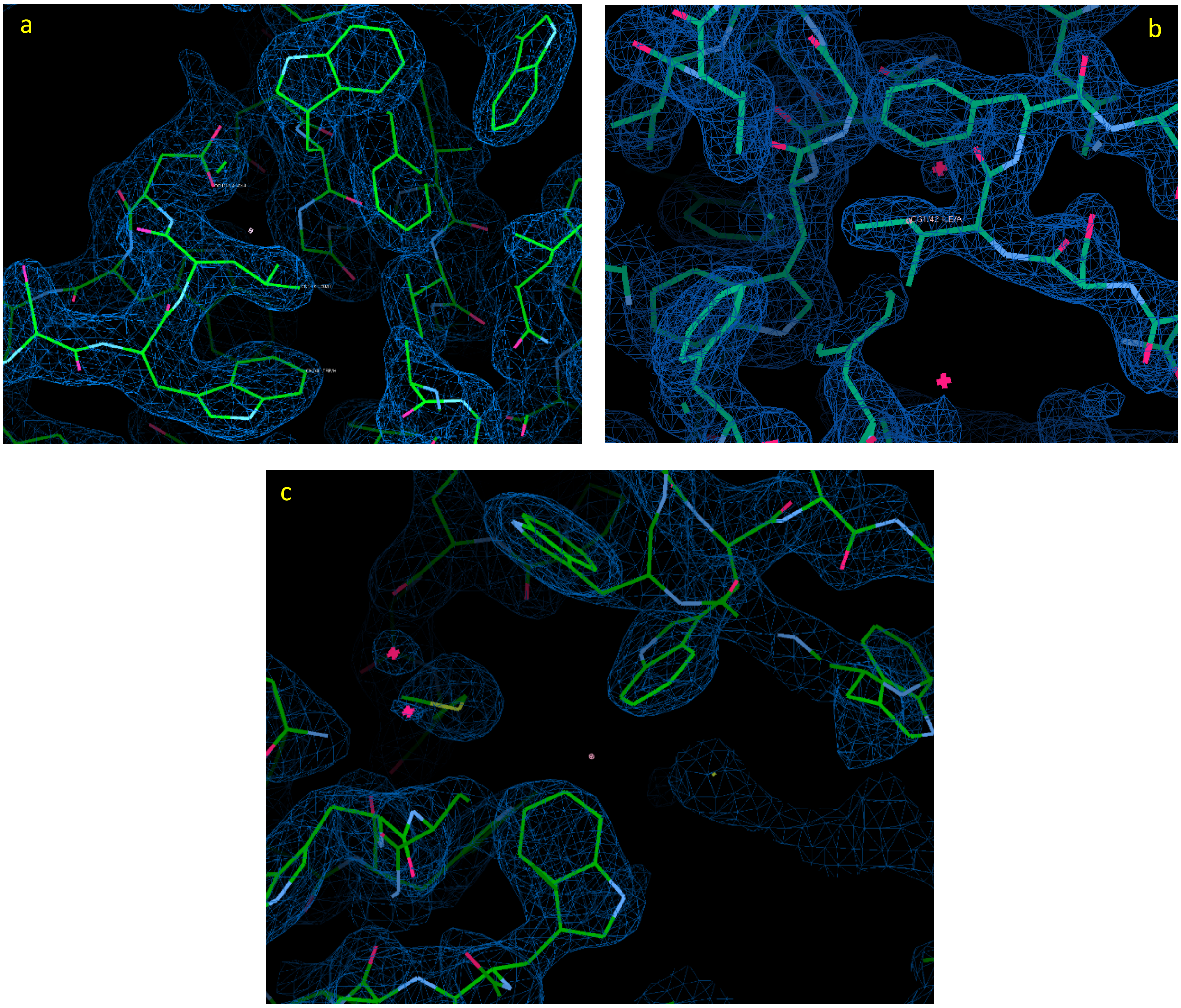

Figure S4. Sample 2Fo-Fc electron density at the final stage of refinement for crystal structures. (a) The interface between Affimer **BCL-x$_L$-AF7** and BCL-xL; (b) interface between Affimer **BCL-x$_L$-AF6** and BCL-xL; (c) interface between Affimer **MCL-AF11** and MCL-1. Despite slightly higher than usual R_{work} and R_{free} (for **BCL-x$_L$-AF7** and **MCL-1-AF11,** probably because of a high overall Wilson B-factor for this crystal), the electron density is absolutely unambiguous. Figure generated using *Coot*[2].

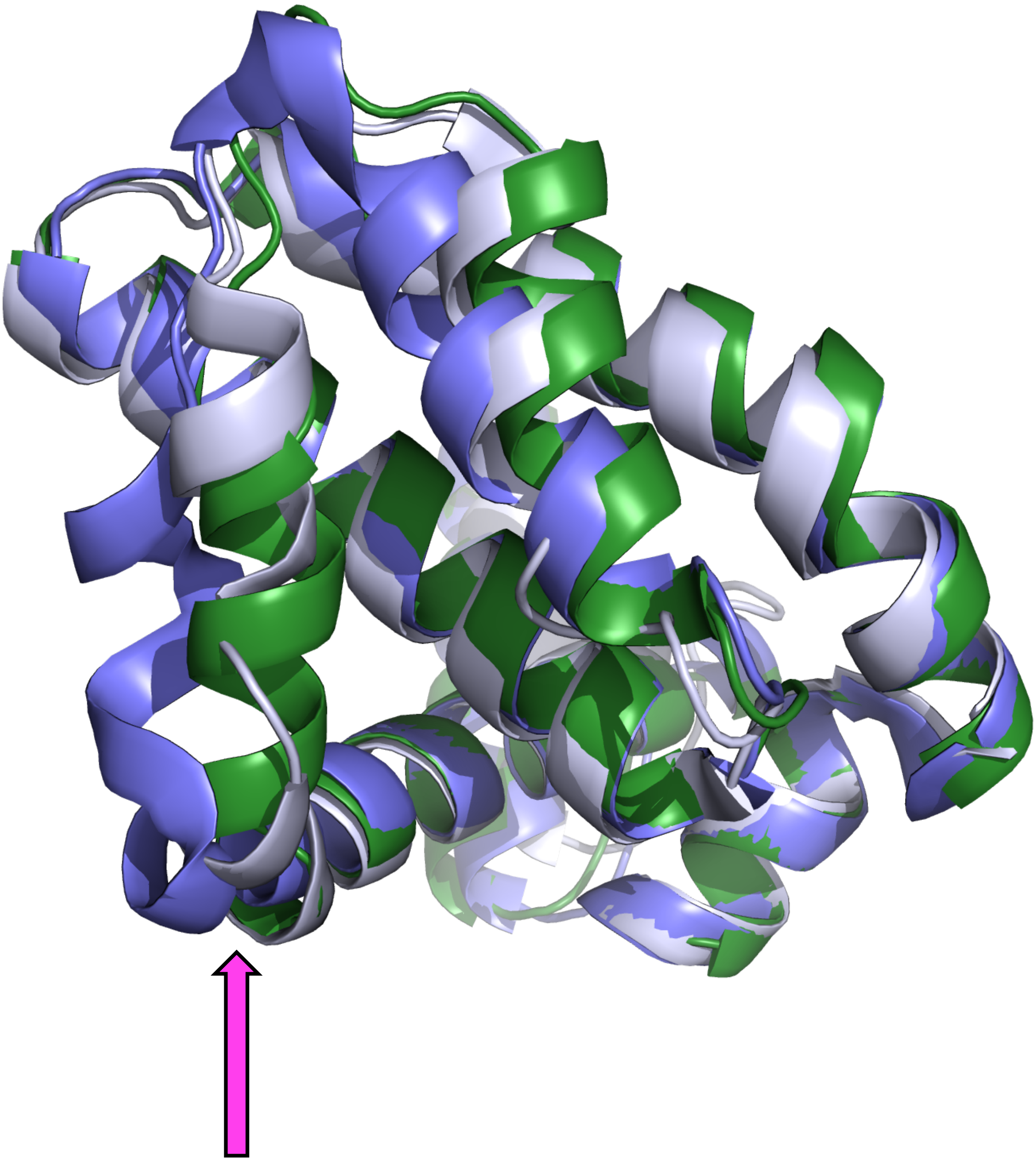

Figure S5. Overlay of Bcl-xL when bound in different complexes. Overlay of Bcl-xL when bound to Affimer (dark green) BIM peptide (purple) or WEHI-539 (light blue). Note the position of the BCL-xL helix to the left of peptide/compound (arrow): the helix binding groove is wider when bound to peptide than to WEHI-539 or BCL-xL-AF7.

References

1. J. A. Miles, D. J. Yeo, P. Rowell, S. Rodriguez-Marin, C. M. Pask, S. L. Warriner, T. A. Edwards and A. J. Wilson, *Chem. Sci.*, 2016, **7**, 3694.
2. A. Oberstein, P. D. Jeffrey and Y. Shi, *J. Biol. Chem.*, 2007, **282**, 13123.
3. D. J. Yeo, S. L. Warriner and A. J. Wilson, *Chem. Commun.*, 2013, **49**, 9131.
4. G. Winter, *J. Appl. Crystallogr.*, 2010, **43**, 186.
5. A. T. Brünger, in *Methods Enzymol.*, Academic Press, 1997, pp. 366.

6. A. J. McCoy, R. W. Grosse-Kunstleve, P. D. Adams, M. D. Winn, L. C. Storoni and R. J. Read, *J. Appl. Crystallogr.*, 2007, **40**, 658.
7. M. K. Manion, J. W. O'Neill, C. D. Giedt, K. M. Kim, K. Y. Z. Zhang and D. M. Hockenbery, *J. Biol. Chem.*, 2004, **279**, 2159.
8. C. Tiede, A. A. S. Tang, S. E. Deacon, U. Mandal, J. E. Nettleship, R. L. Owen, S. E. George, D. J. Harrison, R. J. Owens, D. C. Tomlinson and M. J. McPherson, *Protein Eng. Des. Sel.*, 2014, **27**, 145.
9. P. Emsley, B. Lohkamp, W. G. Scott and K. Cowtan, *Acta Crystallographica Section D*, 2010, **66**, 486.
10. G. N. Murshudov, A. A. Vagin and E. J. Dodson, *Acta Crystallographica Section D*, 1997, **53**, 240.
11. A. A. Vagin, R. A. Steiner, A. A. Lebedev, L. Potterton, S. McNicholas, F. Long and G. N. Murshudov, *Acta Crystallographica Section D*, 2004, **60**, 2184.
12. V. B. Chen, W. B. Arendall, III, J. J. Headd, D. A. Keedy, R. M. Immormino, G. J. Kapral, L. W. Murray, J. S. Richardson and D. C. Richardson, *Acta Crystallographica Section D*, 2010, **66**, 12.
13. .
